# Environmental impact assessment of the coal yard and ambient pollution

**DOI:** 10.1007/s11356-024-32490-z

**Published:** 2024-02-17

**Authors:** Marek Kucbel, Helena Raclavská, Karolina Slamová, Michal Šafář, Barbora Švédová, Dagmar Juchelková, Jana Růžičková

**Affiliations:** 1 CEET/ENET Centre, VŠB-Technical University of Ostrava, 17. listopadu 15/2172, 708 00 Ostrava-Poruba, Czech Republic; 2 Institute of Foreign Languages, VŠB-Technical University of Ostrava, 17. listopadu 15/2172, 708 00 Ostrava-Poruba, Czech Republic; 3 Department of Electronics, Faculty of Electrical Engineering and Computer Science, VŠB-Technical University of Ostrava, 17. listopadu 15/2172, 708 00 Ostrava-Poruba, Czech Republic

**Keywords:** Coal Yard, UAV, Vertical Distribution, Air Pollution, Particulate Matter, Black Carbon

## Abstract

This study investigates the vertical distribution of pollutants emitted from coal yards using unmanned aerial vehicles (UAVs). Vertical concentration measurements of black carbon (BC) and particulate matter (PM) in a range of 1 m to 100 m above ground level (AGL) in the central coal yard showed clear spatial patterns and gradients of these pollutants. In addition, measurements were taken at specific heights (1 m, 30 m AGL, and 60 m AGL) at seven locations approximately 3 km from the yard. Thirteen measurements were carried out during the non-heating period under similar weather conditions. The measured BC concentrations decreased significantly with increasing altitude, with ground-level concentrations reaching 1.88 ± 0.61 µg/m^3^ and decreasing by over 46% at 80 m AGL. Similarly, PM_10_ concentrations at 60 m AGL decreased by 21.7%, with values of 25.99 ± 9.24 µg/m^3^ measured near the ground level and 16.52 ± 8.31 µg/m^3^ at 60 m AGL. The maximum coal particle pollution from the coal depot ranges from 500 to 1,000 m. The study showed a significant decrease in BC concentrations with height above the coal yard surface. Concentrations of PM_10_ and PM_10_-TSP showed a complex distribution influenced by local emissions and long-range particle transport. Meteorological factors, especially wind speed and direction, significantly influenced the pollutant dispersion. In addition, higher pollutant concentrations were measured during dry periods than after rainfall. The findings of this study contribute to a better understanding of the dispersion patterns and potential impacts of coal dust, enabling the implementation of targeted mitigation strategies and improved pollution control measures.

**Supplementary Information:**

The online version contains supplementary material available at 10.1007/s11356-024-32490-z.

## Introduction

Despite the increasing share of renewable energy sources in the global energy mix, coal remains an important fuel and feedstock for some industries. When coal is stored in coal yards in the open air, the effect of wind can lead to the dispersion of fine-grained particles (Wang et al. 2020), which is significant mainly at higher wind speeds (Cheng et al. 2021; Duan et al. 2023). The coal yard acts as a fugitive emission source (Woo et al. 2023), and there are economic losses (Wang et al. 2020). It is estimated that about 30% of coal particle emissions from wind erosion come from wind movement across the coal yard, 40% from the activity itself at the coal yard (coal loading and unloading), and 30% from the operation of trucks at the coal yard (Kurniawan et al. 2021).

The size of coal dust particles released from landfills ranges from < 2 μm to > 100 μm (Larson 2015). Any particles released without a size restriction are referred to as total suspended particles (TSPs). TSPs can be divided into particles > 10 μm as well as particles < 10 μm (PM_10_), particles < 2.5 μm (PM_2.5_), and particles < 1 μm (PM_1_). Coal dust particles from landfills consist of particles containing coal mass (carbon particles containing not only carbon but also H, N, S, and O) and ashes formed by minerals (mainly silicates, carbonates, and sulphides) (Ostro et al. 2023).

In the air, carbon particles are present in PM, which are formed by black carbon (BC) or elemental carbon (EC) together with organic carbon (OC). BC particles are the product of the imperfect combustion of biomass and fossil fuels from local boilers, transport, industrial sources, and power plants (Wyche et al. 2020). Black carbon has adverse impacts not only on human health, particularly the respiratory (Nducol et al. 2021) and cardiovascular systems (Rovira et al. 2022), but also on the environment, where it reduces visibility and absorbs sunlight (Nducol et al. 2021). In urban European air, BC contributes to 5–15% of mass concentrations of PM (Cavalli et al. 2016).

PM_10_ emissions in the coal yard operations are influenced by dust reduction measures and meteorological conditions but also by the intensity and technology used in coal loading/unloading (Kim et al. 2020), wind erosion from coal deposits and coal particle re-suspension (Rojano et al. 2016). The spatial distribution of the particles is influenced by the coal heap height, grain size composition, and petrographic type of coal (Kurniawan et al. 2021), moisture in the surface layer of coal (Techarat and Tontiwachwuthikul 2020), as well as operations on unpaved surfaces and maintenance of local roads (Rojano et al. 2016). Results of emission factors (EF) determined according to the US EPA methodology showed that PM_10_ from coal yards represents about 4.71E-05 g/m^2^s. Of this, 27% is due to emissions generated by wind erosion and 73% is related to activities such as coal loading and unloading and operations on unpaved surfaces around coal deposits (Rojano et al. 2016). Jha and Muller (2017) found that a 10% increase in coal reserves (number of deliveries) results in a 0.06% (0.12%) increase in average levels of PM_2.5_ concentrations within a radius of about 40 km in the wind direction from the place of origin. This increase in PM_2.5_ concentrations brings a risk to the local population in the direction of the airflow from the coal deposit and to areas and periods with lower precipitation (Jha and Muller 2017). Pollution caused by coal dust has a considerable impact on adjacent housing, residents’ health, productivity, and quality of life (Jin et al. 2022). The dust dispersion from open coal yards has become a significant issue that needs to be addressed with appropriate attention (Duan et al. 2023).

Horizontal and vertical air pollutant concentration profiles are significant for understanding air pollutant behaviour regarding air quality prediction. Different methods are used for vertical measurement of pollutant profiles, and the use of UAVs (Unmanned Air Vehicles), commonly known as drones (Lee et al. 2022), is growing in popularity. The availability of UAVs has improved significantly due to rapid technological progress (Poormorteza et al. 2022), low prices, access to hazardous and inaccessible areas and a very user-friendly interface. These features make UAVs an attractive tool for scientific research in various areas (Giordan et al. 2020; Poormorteza et al. 2022). There are obstacles to the adoption and standardisation of measurements using drones, such as limited operating hours and the payload capacity of drones. In addition, the rotary-wing propellers of a drone generate a so-called downwash, which can negatively affect the quality of the measured data. However, chemical monitoring systems based on UAVs are an excellent alternative or complement to traditional ground techniques (Burgués and Marco 2020).

The UAV platform is stable, safe and capable of providing high-resolution three-dimensional measurements of air pollutants and meteorological parameters (Samad et al. 2022). Concentrations of BC up to 100 m from the ground usually decrease with height (Chilinski et al. 2016). Vertical profiles from 0 to 120 m above ground level (AGL) confirmed higher concentrations of air pollutants at the surface due to the proximity of emission sources. PM concentrations decreased with height, and PM_2.5_ decreased by 0.2 μg/m^3^ per 10 m. BC concentrations had a variety of vertical profiles with a total decrease of 0.1 μg/m^3^ per 10 m (Liu et al. 2020; Samad et al. 2022). In addition to local emissions, meteorological parameters play an important role in the vertical spread of PM and BC, particularly relative air humidity, wind direction and speed, atmospheric stability, and regional transport of air pollutants (Liu et al. 2020; Samad et al. 2022). Temperature inversion also influences the increase in ground BC concentrations (Chiliński et al. 2018). The vertical distribution of PM varies significantly depending on the time of year and the time of day, but in most cases, the PM concentration decreases with increasing height from the surface (Liu et al. 2021b; Dubey et al. 2022b, a). In addition to meteorological factors, the PM concentration is significantly influenced by the height of the boundary layer (Jin et al. 2020).

Most previous studies have focused on research and monitoring of PM in the ground layer. These studies have provided information on the horizontal distribution of particulate matter (PM) and other pollutants on the surface. However, in order to understand the spread of air pollution better, it is essential to understand the vertical distribution of concentrations of pollutants. Most of the previous research on the environmental impact of landfills has dealt with the issue of particle distribution in terms of size: PM_2.5_, PM_10_, and TSP (Blackwood and Wachter 1978; Davis and Boegly 1978; Mueller et al. 2015a, b; Kim et al. 2020; Woo et al. 2023). The studies are mainly focused on the properties of air pollutants and the variables influencing them in horizontal ground measurements. There is a lack of information on the vertical dispersion of air pollutants from coal yards, which can significantly influence the overall range of pollution from a fugitive source.

This study uses modern approaches to assess the vertical distribution of dust particles from coal yards and their impact on the environment in urban agglomerations. The results presented in this paper provide completely new and unique insights into the vertical dispersion of coal particles and enable the determination of the emission impact of coal yards on the environment. This important information from coal yards was not available in the previous literature and has not been analysed using this method before.

## Materials and methods

The UAV DJI Matrix 600 Pro (DJI Technology Co., Shenzhen, China) with dimensions of 1,668 mm (rotor diameter) × 727 mm (height), weighing 9.5 kg and carrying capacity of up to 6 kg was used for vertical measurements. During the measurements, the drone ascended vertically from the ground to 60 m at a constant rate of 2 m/s. The measurements occurred at ground level (1 m), 30 m, and 60 m above ground level (AGL) for 180 s. The UAV was operated in accordance with the legislative requirements for safe civilian use of drones in each category and subcategory (as open, specific, or certified) under the EU Easy Access Rules for Unmanned Aircraft Systems (Regulations (EU) 2019/947 and 2019/945) (European Commission 2019a, b).

### Method of determination of the concentration of dust particles and black carbon

The elevation profile was obtained by measuring dust particles with the Fidas® Frog (Palas, Karlsruhe, Germany) and the BC aethalometer MicroAeth/MA2000 Aethlab, San Francisco, USA). In order to minimise the effect of air turbulence caused by rotor action, the Tygon® Teflon sampling hose with a length of 1,500 mm is used for the measurements on both instruments. The measured data was stored in an internal memory from which it was downloaded after the measurements were completed. The data was measured at the climb of the UAV.

The Fidas® Frog optical counter was used to measure the concentrations of the dust particles. The device is able to measure the concentrations of PM_1_, PM_2.5_, PM_4_, PM_10_, and TSP, as well as the number of particles at the same time. The measurement range for the mass concentration ranges from 0 to 100 mg/m^3^; for the numerical concentrations, it is 0 to 20 × 10^9^ particles/m^3^. This device works with a volume flow rate of 1.4 L/min (Palas 2023). From the point of view of monitoring and assessing the impact of pollution from coal yards, PM_10_ and PM_10_-TSP have proved to be the key coarse-grained dust particles. For this reason, we focused on the detailed characteristics of these above-mentioned dust particles, which provide detailed information on their composition, origin, and distribution in the air. From the point of view of assessing the composition of ∑TSP, the dust particles were divided into grain size classes: PM_<1_, PM_1-2.5_, PM_2.5–10_, and PM_10_-TSP.

The principle of determining the concentration of black carbon (BC) is to measure the attenuation of a beam of light passing through a quartz fibre filter, on which aerosol particles are continuously captured. MicroAeth/MA200 was used to measure BC; it is suitable for compact UV-IR BC monitoring with five wavelengths (375, 470, 528, 625, and 880 nm) with an automatic filter tape displacement system. It is also equipped with sensors for recording GPS, temperature, and relative humidity. For quantitative and qualitative determination of BC, concentrations obtained from a near-infrared channel (880 nm) of a micro-aethalometer were used (AethLabs 2018). The selected wavelength of 880 nm corresponds to the quantitative BC value, while the influence of other light-absorbing aerosols is negligible (Hansen 2005). The detection limit of MA200 is 30 ng/m^3^ at a time resolution of 1 s, with a flow rate of 150 mL/min (AethLabs 2018). The data recording interval for MA200 was set to 10 s. This interval was chosen deliberately due to the absence of negative values in the 10-s time interval following optimised noise reduction. The averaging processing indicates that a satisfactory smoothing effect is achieved for black carbon concentrations (Liu et al. 2021a).

Meteorological parameters were measured by a professional digital weather station at a ground level located in the town of Karviná, operated by the Health Institute based in Ostrava.

### Software

Statistical analysis for descriptive statistics, correlation analysis, and the one-way analysis of variance (ANOVA) was performed using the statistical software OriginPro 8.5 and Origin Pro2019. For correlation analysis, the Spearman correlation coefficient at the level of significance α = 0.05 was used. The hierarchical cluster analysis (HCA) was performed using the statistical software Statgraphics Plus 5.1 (Statistical Graphics Corp.).

For constructing the model of isolines of two monitored variables at three elevation levels, the program Surfer, version 2021 (Golden Software, Inc.) was used. Input data were subjected to exploratory data analysis. The Kolmogorov–Smirnov test and Shapiro–Wilk test at the level of significance α = 0.05 were used to test the normality of data sets. The model was constructed using kriging (linear model of variogram, without drift). The most suitable model was selected based on the results of cross-validation.

### HYSPLIT model

HYSPLIT backward trajectories were used to study air mass transport, long-range transport, dispersion, and source of air pollutants. The backward trajectories were modelled in the HYSPLIT (Hybrid Single Particle Lagrangian Integrated Trajectory Model) program provided by the NOAA Air Resource Laboratory (Stein et al. 2015; Rolph et al. 2017). It is a Lagrangian model that primarily works with the prevailing values for wind direction and strength at the time of measurement. The Global Assimilation System (GDAS) model, with a 1° × 1° resolution, was used to obtain the meteorological data. The output of the model is a map of the path of the air mass towards the location under investigation. The starting point for measuring the backward trajectories was the geographical centre of the study area (centre of the coal yard: latitude: 49°49′26.7" N, longitude: 18°29′02.1" E). The return trajectories were modelled for single measurements in 2022 for 24 h at 100 m above ground level (AGL).

### Site description

**Fig. 1:**
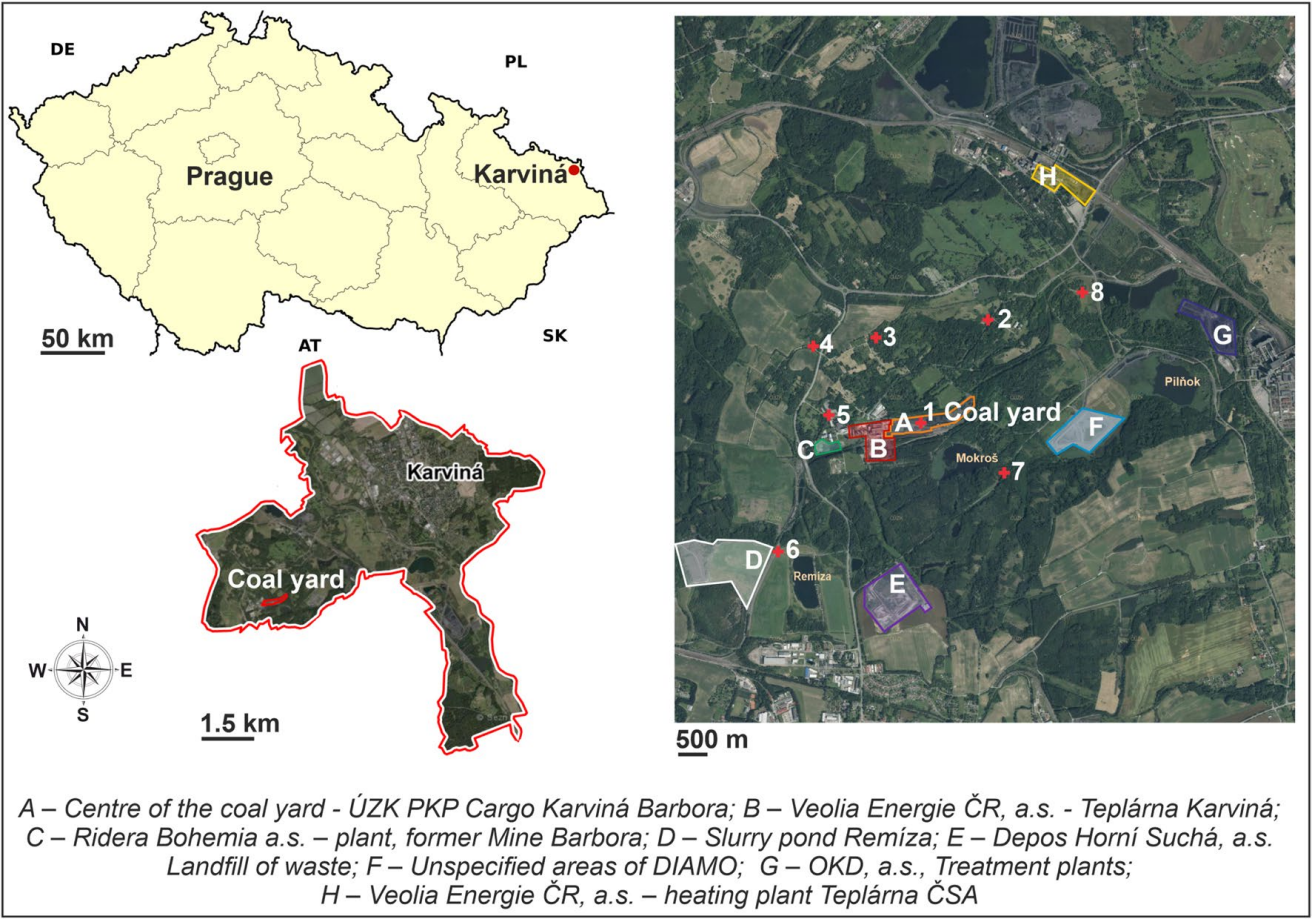
The position of the measured locations in the topographical map

The studied area (Fig. 1) is the coal yard PKP Cargo Karviná Barbora (Karviná-Doly, Moravian-Silesian Region, Czech Republic) is situated in the area of the logistics and distribution centre Upravárenský závod Karviná (ÚZK) located in the area of the former Barbora Mine. The facility was built in 2004–2005. The area is situated in a locality strongly influenced by mining activities between the towns of Karviná and Havířov in the Ostrava-Karviná coal basin. In the immediate vicinity of the studied locality, there is the heating plant Teplárna Karviná (Veolia Energie ČR), administrative buildings and production sites, which served as the background of the mine. In the vicinity, there is a situated Ridera Bohemia facility dealing with recycling and sales of inert materials. Other potential dust sources include the heating plant Teplárna ČSA (Veolia Energie ČR). Total emissions of solid pollutants (TZL) weighing 6,694 tonnes (Czech Hydrometeorological Institute 2023a) were reported from the heating plant Teplárna ČSA for the year 2021 and 7,803 tonnes (Czech Hydrometeorological Institute 2023b) from the heating plant Teplárna Karviná. The area of interest also includes the company Depos Horní Suchá, which operates the waste dump (used for the category of other wastes). Finally, there are the Remíza Pond, the Pilňok Pond, and other areas owned by the state enterprise DIAMO without any further specification of their use. This location thus provides an ideal area for monitoring the distribution of coal particles in the vicinity of the coal yard. The nearest residential development is located about 600 m from the site. The deposition of contaminants at the locality is influenced by significant sources of pollution in the agglomeration, in winter by the long-distance transmission of air pollution from Poland, and locally by local sources (home heating in winter).

The Barbora coal yard covers an area of about 0.85 km^2^ with a maximum capacity of 200,000 tonnes of fossil fuels (bituminous coal + coke). At the time of the measurement, about 41% of the capacity of the coal yard was used. The coal yard contains bituminous coal from the Upper Silesian Coal Basin (Poland and Northern Moravia – Karviná) and the USA (Appalachian Coal Basin, West Virginia, Beckley Mine with different grain sizes from < 10 mm to 100 mm). The coal yard, where fossil fuels – coke and coal are stored, uses measures to reduce dust particles, such as spraying the site with tanker lorries and, in extreme conditions, the use of a fogging machine. Dust control measures include limited lorry speeds, reduced drop heights for material handling and fogging equipment, etc.

**Fig. 2:**
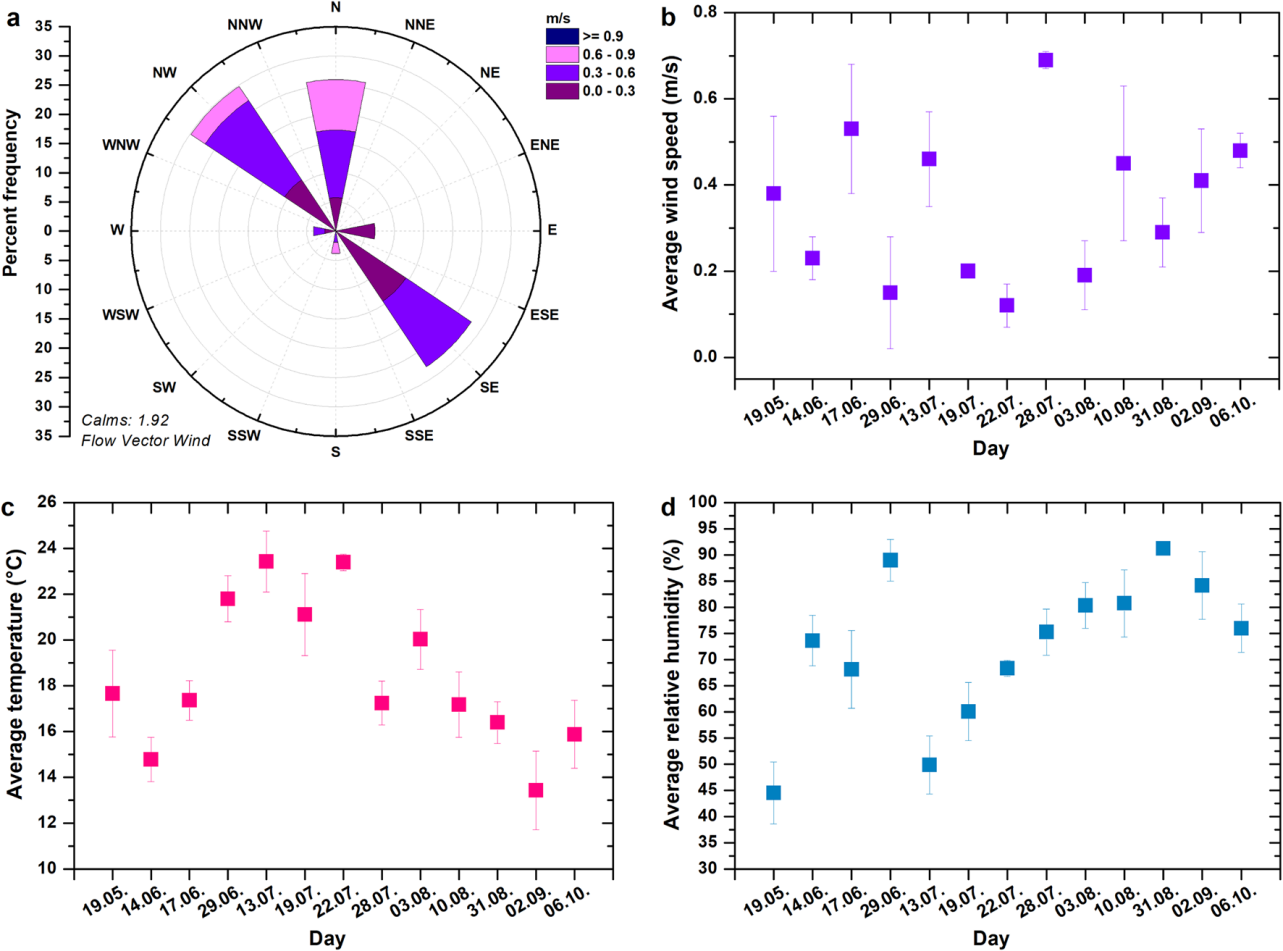
Wind rose for the coal yard area during the measurements carried out in 2022 (n = 13) (**a**); basic meteorological parameters recorded during the measurements in 2022 (n = 13) – average wind speed (**b**); average temperature (**c**); average relative humidity (**d**)

The vertical distribution of black carbon and PM around the coal yard was monitored during 2022 (Table 1 and Fig. 1). Individual measurements (n = 13) were carried out in the morning (from 9:00 to 12:00) between May and October 2022. The measurements were carried out altogether at eight sites: four times in July, three times in June and August, and once in May, September, and October. The periods were chosen to reflect similar meteorological conditions and suitable conditions for UAV flight. The individual measurement days in 2022 are visualised in Fig. 2. Based on the results of the 2021 measurements, the following altitudes were selected to monitor the vertical distribution of particles at eight sites: ground level, 30 m, and 60 m AGL. In 2021, the vertical distribution of black carbon, PM_10_ and PM_10_-TSP was monitored at altitudes 1 (ground level), 10, 20, 30, 40, 60, 80, and 100 m above the surface of the coal heap (AGL). The measurements of the vertical distribution of coal and dust particles were carried out only at the centre of the coal yard area (site 1) from February to November 2021 with a monthly frequency (10 measurements). The measurements were carried out under favourable dispersion conditions, allowing the use of UAV.

**Table 1:** Measuring sites for the study area with GPS coordinates

Site number	1	2	3	4	5	6	7	8
GPS_Lat	49.824085	49.832466	49.830391	49.829307	49.824724	49.813482	49.82087	49.835078
GPS_Lon	18.483913	18.491069	18.478053	18.470752	18.472962	18.468511	18.494456	18.501574

At the site of ÚZK PKP Cargo, Barbora, all-year-round (365 days) conditions of very mild flow prevail with wind speeds of 0–5 m/s with prevailing SW, SE, and E wind directions. During the measurements made in 2022 (13 measurements), NW, SE, and N wind direction prevailed (Fig. 2). The average air temperature at which measurements were made was 18.43 ± 3.34 °C, wind speed 0.35 ± 0.19 m/s, relative air humidity 72.41 ± 14.47%, and air pressure 1024.3 hPa.

## Results and discussion

The measurement results obtained from the sensors and mobile analysers attached to the UAV depend fundamentally on the position of its location on the drone, the type of UAV used, the characteristics of the load used (e.g., weight) and the target use (Burgués and Marco 2020). The most suitable location is the isolation of the sensor technique for detecting air contaminants in the lower airflow from the rotor propellers, e.g., by extending the sampling tube (Falabella et al. 2018). Accurate data was obtained during the altitude measurement with the UAV during the ascent, but during the descent, the concentrations were about 50% overestimated (Hedworth et al. 2022). In order to ensure accuracy and efficiency, it is recommended during the vertical measurement to collect only the data at the UAV exit and, at the same time, to place the suction tube under the centre of the UAV to avoid stability problems.

### *Vertical distribution of air pollutants from 1 to 100 m AGL over the centre of the coal heap and selection of suitable height for further measurements*

**Fig. 3:**
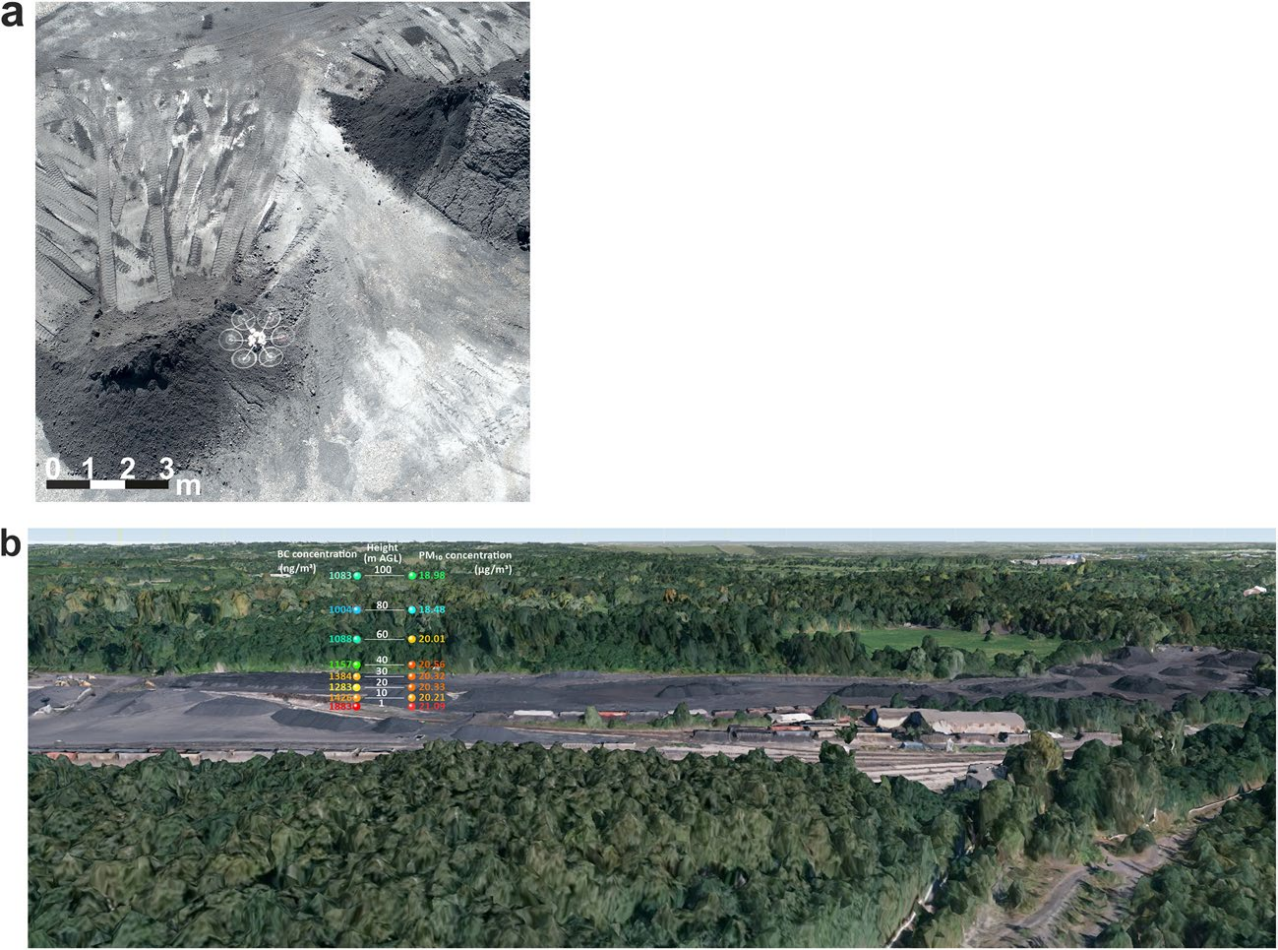
(**a**) Demonstration of UAV DJI flight over a coal heap body; (**b**) Average concentrations of BC particles above the centre of the coal heap body

For vertical measurements carried out in 2021 (Fig. 3) only at site 1 (coal yard ˗ CY), the centre of the coal heap (10 measurements) at ground level (1 m AGL) to 100 m AGL, statistically significant polynomial dependencies were found between height and concentration of BC (r = 0.93, α = 0.05), PM_10_ (r = 0.93, α = 0.05), and PM_10_-TSP (r = 0.83, α = 0.05). For the determination of the differences in concentrations of air pollutants (BC, PM_10_ and PM_10_-TSP) between individual heights (ground level to 100 m AGL) and in individual months, they were verified using the one-way ANOVA method. Differences between the measured values were confirmed only on the days of the occurrence of maximum and minimum concentrations. The maximum concentration was measured at the time of operation of intensive heavy equipment at the coal yard.

The TSP was dominated by coarse-grained particles of the grain size class PM_10_-TSP, which, on average, accounted for 56.37 ± 11.34% of the ∑TSP at all monitored heights of 1–100 m AGL. In contrast, minimal concentrations of air pollutants were demonstrated during calm airflow. In this case, the ∑TSP had a high proportion of fine-grained PM particles < 1 µm, which was 80.62 ± 4.58% at the monitored height of 1–100 m AGL; it corresponds to the origin of the particles from long-distance transport.

**Fig. 4:**
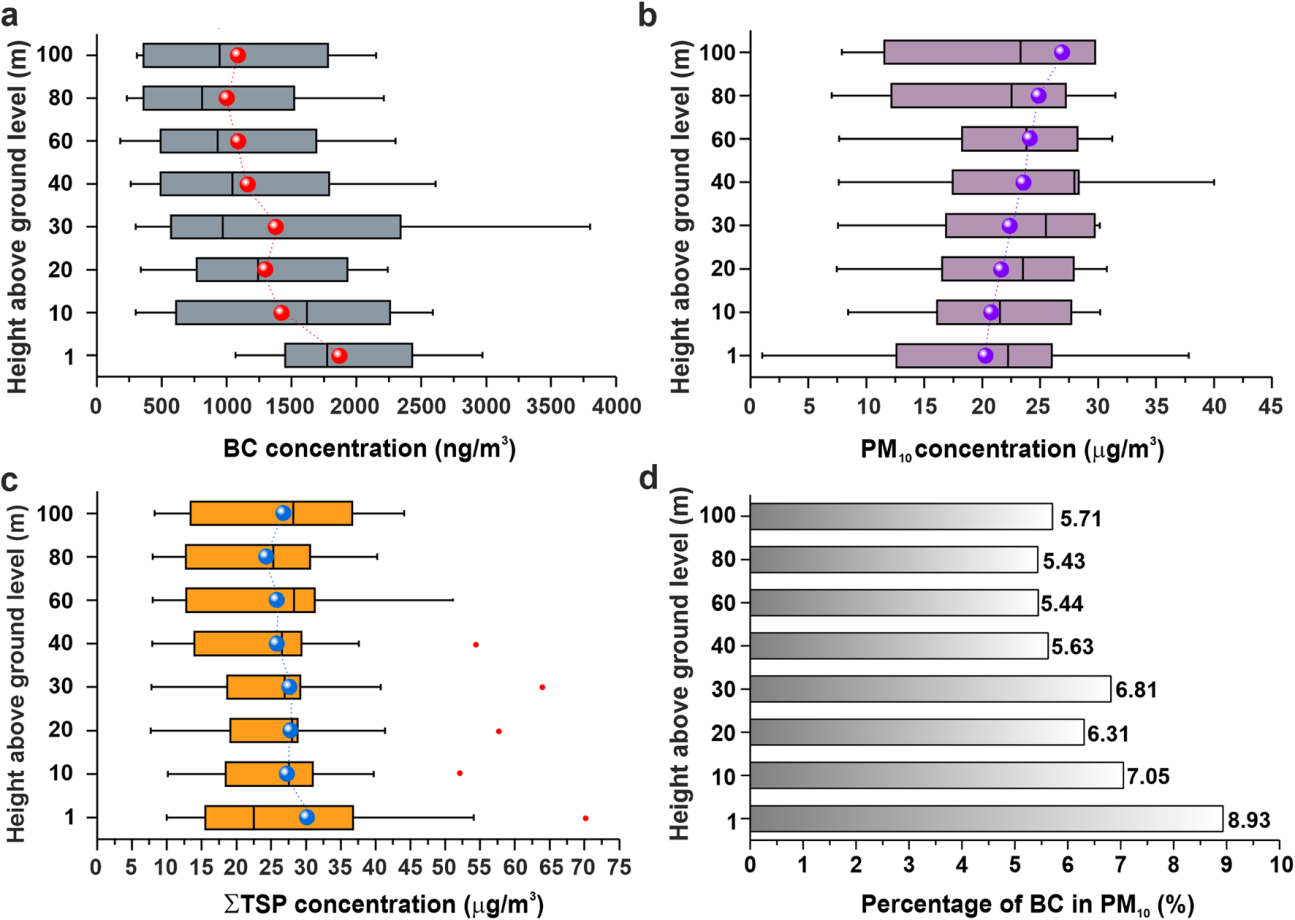
(**a**) Box plot for vertical distribution of average black carbon concentrations; (**b**) PM_10_; (**c**) ∑TSP; (**d**) percentage of BC in PM_10_ measurements at the centre of the body of the coal heap at altitudes of 1 to 100 m AGL between February and November 2021

The average concentration (10 measurements) of BC particles decreases with increasing height from the coal surface (Fig. 4). The highest average concentration of BC particles was found for ground level 1,883 ± 613 ng/m^3^. In contrast, the lowest concentration (1,004 ± 720 ng/m^3^) was measured at 80 m AGL. The difference between the highest and lowest BC concentration was 879 ng/m^3^; the decrease in BC particle concentration was thus more than 46% at 80 m AGL.

The highest average concentration of PM_10_ (21.09 ± 10.38 µg/m^3^) was found at ground level, with concentrations decreasing slightly as the height from the surface increased. The lowest average concentration of PM_10_ was found at 80 m AGL at 16.52 ± 8.31 µg/m^3^, representing a 21.7% decrease in PM_10_ concentration compared to ground level, with the average concentration of PM_10_ increasing again at 100 m AGL to 19.80 ± 8.81 µg/m^3^.

In the case of PM_10_-TSP, the highest average concentrations for the ground level were also found (8.88 ± 10.85 µg/m^3^). Up to 60 m above the surface of the coal heap, the TSP concentration decreases by approximately 34% from the ground level. From a height of 80 m, the PM_10_-TSP concentration increases again (Fig. 4). The distribution of PM_10_ and PM_10_-TSP particles depending on height has a polynomial character, characterised by a decrease in particle concentration from the ground level to 60 m AGL, followed by an increase caused by another source predominantly of inorganic particles or by long-distance transport. These results correspond to the findings reported by Samad et al. (2022). Hakala et al. (2022) found that anthropogenic air contaminants mostly occur at ground level and are trapped within the boundary layer, particularly in periods shorter than one week.

For the area of interest, it was found that the amount of PM_10_ and PM_10_-TSP decreases with height, which is in line with the results found for PM_10_, PM_2.5_, and PM_1_ (Deng et al. 2015). Monitoring of the distribution of PM_10_ by balloon over fugitive emission sources (tailings, landfills) in the Ostrava region revealed that for most fugitive sources, such as tailings and landfills, the increased concentration of PM_10_ occurs at the height of 30 to 40 m above the source (Štrbová et al. 2017). The increase in concentration may be influenced by the presence of an inversion layer, which is formed at an altitude above 350–365 m above sea level, which corresponds to an altitude of 123–138 m AGL (Štrbová et al. 2017). Monitoring of PM_10_ concentration by UAV in Krakow (Poland) showed three vertical zones of potential air pollution (Sekuła et al. 2021). The first zone (up to approximately 60 m above sea level) has the worst pollution conditions, which occur during periods of low wind speed when air pollution is potentially highest and lasts for a long time. The second zone, between 60 and 100 m above sea level, is a transitional zone with high levels of PM_10_, but a significant decrease is observed with height. The third zone (above 100 to 120 m above sea level) has significantly better air quality than the first zone, which may be due to increased wind speed, wind direction changes or advection of clean air masses (Sekuła et al. 2021).

The results of the UAV measurements in 2021 showed that the coal yard affects the occurrence of BC particles up to a height of up to 30 m and most significantly at ground level, with BC concentrations in PM_10_ representing around 9% (Fig. 4). For this reason, the heights of 1 m (ground level), 30 m, and 60 m AGL were selected for the 2022 measurements.

From individual measurements, the correlation between the concentration of BC and PM_10_ was calculated for individual heights. A significant correlation between BC and PM_10_ was demonstrated only for the height of 10 m AGL, where the BC content of PM_10_ is highest.

### Detailed analysis and impact on the vertical distribution of dust particles over the coal yard and surrounding area

#### Geochemical background and anomalies

The geochemical background and geochemical anomaly threshold values for BC, as well as PM_10_ and PM_10_-TSP dust particles, were calculated for the 2022 assessment of the environmental load in the coal yard area (Table 2). The geochemical background value represents the arithmetic mean of all measured values for the whole monitored area. The geochemical anomaly threshold was determined as the geochemical background value increased by twice the standard deviation value according to ISO 19258:2018 “Soil quality – Guidance on the determination of background values”. The statistical significance of the differences in the concentration of air pollutants in the coal yard and its wider surroundings was monitored by one-way ANOVA. The significance of the differences in concentration of BC, PM_<1_, PM_1-2.5_, PM_2.5–10_, and PM_10_-TSP at 1, 30 and 60 m AGL between sites was assessed. The differences in concentrations of air pollutants at 1, 30, and 60 m AGL between sites on one sampling day and on all sampling days were also evaluated. The results of one-way ANOVA did not show a difference between site No. 1 (coal yard) and seven other nearby sites for BC concentrations or PM.

**Table 2:** Values of geochemical background and anomaly threshold

	BC			PM_10_			PM_10_-TSP		
	(ng/m^3^)			(µg/m^3^)			(µg/m^3^)		
AGL	GL	30 m	60 m	GL	30 m	60 m	GL	30 m	60 m
Geochemical background	1125.47	1123.19	1078.94	23.24	23.42	22.85	16.36	15.02	15.37
Standard deviation	329.72	322.03	323.37	9.14	9.79	9.64	11.19	10.02	10.73
Geochemical anomaly threshold	1784.91	1767.25	1725.68	41.51	42.99	42.13	38.74	35.06	36.84
Minimal value	348.17	405.58	355.42	8.67	7.78	6.58	0.01	0.01	0.01
Median	1134.67	1127.17	1091.63	20.60	21.16	20.57	18.94	16.01	16.21
Maximum value	1900.08	1897.83	1702.33	38.97	43.31	40.15	43.46	40.60	41.11

Differences in geochemical background values for both BC and PM concentrations at different heights are negligible (Table 2).

#### ***Average concentrations of air pollutants depending on height for individual locations***

**Fig. 5:**
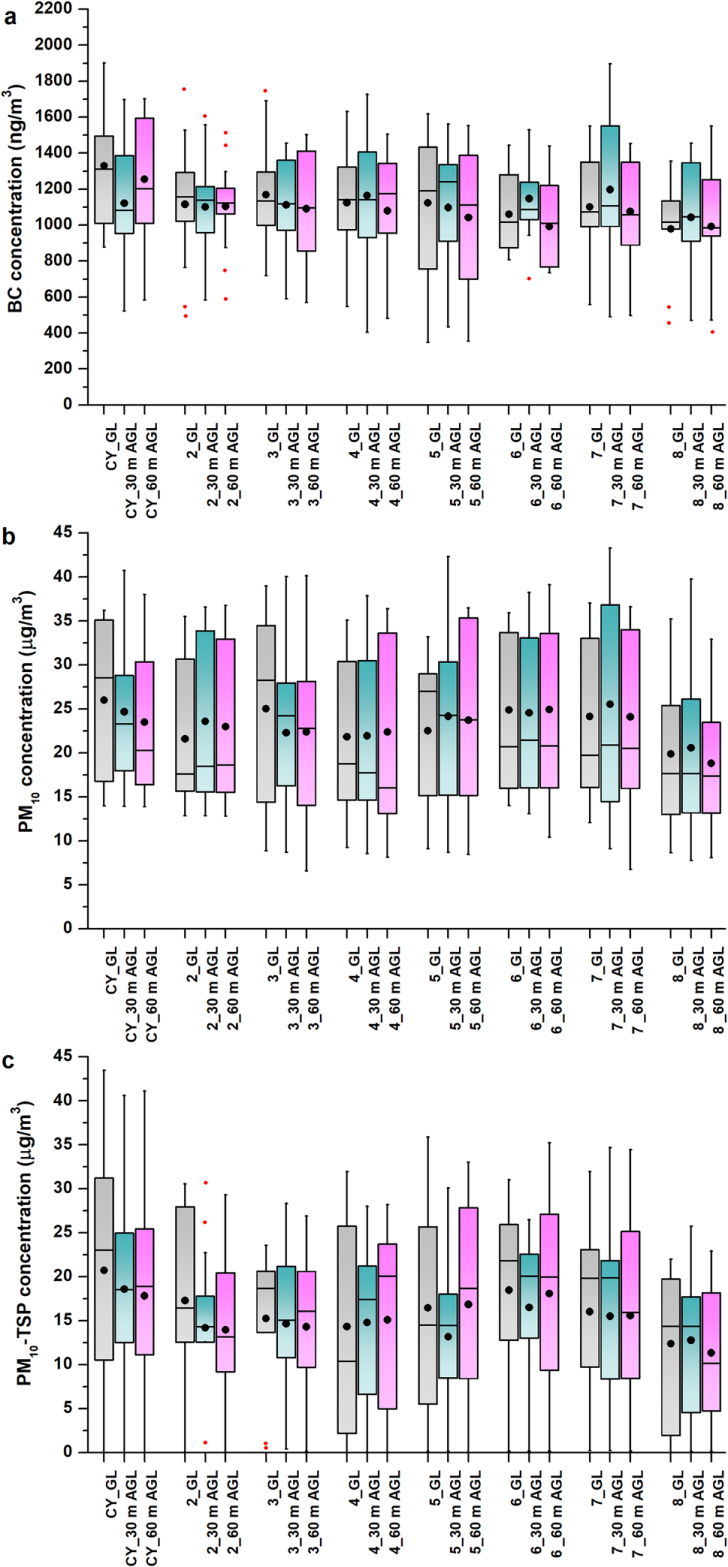
(**a**) Box plot for concentrations of BC; (**b**) PM_10_; (**c**) and PM_10_-TSP at ground level, 30 m AGL, 60 m AGL above the body of the coal yard and for sites in the surrounding area

A comparison of the values of BC, PM_10_ and PM_10_-TSP (Fig. 5) for the body of the coal heap (CY) and for the sites in the vicinity (sites number 2–8) shows that the average BC concentrations above the coal yard at ground level (1,330 ± 322.8 ng/m^3^) are 17.6% higher than at the sites in the vicinity (1,096 ± 322.2 ng/m^3^). A similar trend can be seen at the height of 60 m AGL, where BC concentrations above the coal yard (1,255 ± 360.1 ng/m^3^) were 16.1% higher than at the other sites (1,053 ± 314.5 ng/m^3^). Only at the height of 30 m AGL were the average BC concentrations comparable for the coal yard (1,121 ± 351.5 ng/m^3^) and for the sites in the vicinity (1,123 ± 323.5 ng/m^3^). BC concentrations at this height in the area under review reach a comparable value (corresponding to the background value for 30 m AGL) and cannot be linked to coal yard activities.

The average PM_10_ concentrations for the coal yard at ground level (25.99 ± 9.24 µg/m^3^) were about 3.15 µg/m^3^ (12.1%) higher than for other sites in the surrounding area (22.84 ± 9.20 µg/m^3^). The difference between the average PM_10_ concentrations for the coal yard and other sites was less pronounced for a height of 30 m AGL (1.44 µg/m^3^ – 5.9%) and for a height of 60 m AGL (0.74 µg/m^3^ – 3.14%). The most significant difference was observed for coarse-grained particles of the PM_10_-TSP grain size class. The average PM_10_-TSP concentrations at ground level were about 24% (i.e., 4.99 µg/m^3^) higher for the coal yard than for sites in the surrounding area. As the height from the surface increases, this difference decreases to 22% (4.07 µg/m^3^) for 30 m AGL and 15.6% (2.79 µg/m^3^) for 60 m AGL (Table 3). More coarse-grained PM_10_-TSP dust particles contribute to the increased dustiness of the coal heap, which, due to weight, quickly sediment at the site of their formation and are not transported over greater distances.

**Table 3:** Basic statistical parameters of concentrations of PM10, PM10-TSP, and BC particles at individual locations

		GL				30 m AGL				60 m AGL			
		Mean	SD	Min	Max	Mean	SD	Min	Max	Mean	SD	Min	Max
CY_BC	ng/m^3^	1330.10	322.79	877.09	1900.10	1121.25	351.46	521.94	1697.31	1255.26	360.05	584.18	1702.32
CY_PM_10_	µg/m^3^	25.99	9.24	13.97	36.22	24.68	9.48	13.93	40.74	23.49	8.98	13.89	38.00
CY_PM_10_-TSP	µg/m^3^	20.73	14.32	0.01	43.46	18.58	13.06	0.01	40.60	17.81	13.52	0.01	41.11
2_BC	ng/m^3^	1115.29	357.83	494.75	1756.44	1101.01	308.03	583.44	1601.66	1104.43	255.38	589.08	1515.60
2_PM_10_	µg/m^3^	21.60	8.40	12.88	35.52	23.58	9.19	12.86	36.59	22.99	9.03	12.83	36.75
2_PM_10_-TSP	µg/m^3^	17.29	11.36	0.02	30.55	14.20	9.54	0.01	30.58	13.95	9.66	0.01	29.30
3_BC	ng/m^3^	1168.83	338.86	720.15	1748.11	1112.87	282.68	589.66	1456.00	1090.78	308.61	570.74	1502.58
3_PM_10_	µg/m^3^	25.03	11.10	8.87	38.97	22.28	8.56	8.70	40.04	22.40	10.28	6.58	40.15
3_PM_10_-TSP	µg/m^3^	15.26	8.63	0.43	23.56	14.63	9.52	0.43	28.35	14.30	9.33	0.15	26.90
4_BC	ng/m^3^	1124.67	335.59	547.34	1632.91	1164.78*	371.43	405.58	1727.08	1079.88	341.65	480.73	1504.59
4_PM_10_	µg/m^3^	21.83	9.47	9.25	35.07	21.95	10.04	8.58	37.87	22.38	11.21	8.14	36.38
4_PM_10_-TSP	µg/m^3^	14.33	12.23	0.01	31.93	14.77	10.05	0.06	27.98	15.09	10.89	0.05	28.21
5_BC	ng/m^3^	1124.09	427.50	348.19	1618.73	1097.53	373.91	434.41	1560.97	1042.30	413.43	355.41	1552.23
5_PM_10_	µg/m^3^	22.53	8.45	9.11	33.19	24.16	10.58	8.71	42.33	23.71*	10.38	8.47	36.47
5_PM_10_-TSP	µg/m^3^	16.45	12.15	0.10	35.87	13.17	9.30	0.11	30.09	16.84	11.51	0.04	33.00
6_BC	ng/m^3^	1060.11	216.23	807.00	1443.72	1147.75*	229.25	705.81	1529.41	992.02	237.84	734.48	1440.61
6_PM_10_	µg/m^3^	24.88	8.89	14.01	35.93	24.55	9.53	13.10	38.26	24.93*	9.80	10.42	39.11
6_PM_10_-TSP	µg/m^3^	18.46	11.38	0.16	31.02	16.52	9.53	0.16	26.48	18.09*	11.62	0.16	35.24
7_BC	ng/m^3^	1101.75	285.48	558.41	1549.27	1196.82*	364.84	491.90	1897.83	1075.03	280.00	497.75	1452.57
7_PM_10_	µg/m^3^	24.15	9.44	12.09	37.02	25.54*	12.22	9.11	43.31	24.08*	10.27	6.76	36.63
7_PM_10_-TSP	µg/m^3^	16.02	10.61	0.24	31.96	15.51	11.17	0.21	34.68	15.56	11.58	0.19	34.47
8_BC	ng/m^3^	979.01	294.07	455.64	1375.89	1043.57	334.28	470.82	1454.52	991.81	363.89	409.16	1551.12
8_PM_10_	µg/m^3^	19.88	8.67	8.67	35.24	20.59	10.03	7.78	39.79	18.81	8.31	8.10	32.92
8_PM_10_-TSP	µg/m^3^	12.37	8.77	0.05	21.99	12.77	8.96	0.11	25.73	11.33	8.19	0.11	22.92

Explanation: * – concentration of the parameter higher than the average value above the centre of the coal yard

The average PM_10_ concentrations found at the centre of the PKP Cargo coal yard are slightly higher than reported by Mueller et al. (2015a) but are consistent with results published in other studies (Table 4). Measurements of PM_10_ at four sites near an open-pit coal mine in northern Colombia showed that average concentrations were found to be twice as high during the dry season as during the wet and transition period from 14.2 ± 4.8 μg/m^3^ to 55.5 ± 21.2 μg/m^3^ (Arregocés et al. 2022).

**Table 4:** Comparison of PM_10_ concentrations with literature

Area	Site	Season	PM_10_		Ref
			Mean	Max	
Coal yard—Gallatin Power Plant (USA)	Downwind site 2	June-November 2012	14.3	134	^a^
	Downwind site 3	June-November 2012	11.5	146	
	Downwind site 2	June-November 2012 Without activity^*^	19.7		^b^
	Downwind site 3	June-November 2012 Without activity^*^	21.1		
	Downwind site 2	June-November 2012 With activity^*^	60.7		
	Downwind site 3	June-November 2012 With activity^*^	59.9		
Coal yard—UNI Power Plant Iowa (USA)	South-Windward side	July–October 2008	34.49	77.83	^c^
	North-Leeward side	July–October 2008	26.94	48.64	
Coal yard—PKP Cargo,Barbora,Karviná (CR)	Centre-Ground level	May–October 2022	25.99	36.22	^This study^
	Centre-30 m AGL	May–October 2022	24.68	40.74	
	Centre-60 m AGL	May–October 2022	23.49	38.00	

Explanations: *Average upwind and downwind PM_*10*_* concentrations adjusted to the concentration of C*_xs_* for hours without and with human activity in the coal yard (adjusted concentration C_xs_* provided a consistent measure of coal yard impact on downwind particulate levels across all hours); ^*a*^* – *Mueller et al. (2015a)*; *^*b*^* – *Mueller et al. (2015b)*; *^*c*^* – *Wittenburg (2018)

The modelling of dust particles used information on the distribution of PM_10_ concentrations, which are mainly influenced by the operation of coal yards. Directive 2008/50/EC of the European Parliament and of the Council of 21 May 2008 on ambient air quality and cleaner air for Europe, as amended, sets the value of the daily air pollution limit for PM_10_ at 50 μg/m^3^ with a maximum permitted number of exceedances of 35 times per year and the annual average value must not exceed 40 μg/m^3^. None of the sites surveyed exceeded the daily air pollution limit for PM_10_ in a single case. The average PM_10_ concentrations at all sites were around 23 μg/m^3^ at ground level. This value represents about half of the daily air pollution limit allowed. At the same time, this value complies with the recommended average 24-h PM_10_ concentration of 45 μg/m^3^ recommended by the global air quality guidelines of the World Health Organisation (WHO 2021).

#### ***Distribution of air pollutants in the vertical direction and cluster analysis***

**Fig. 6:**
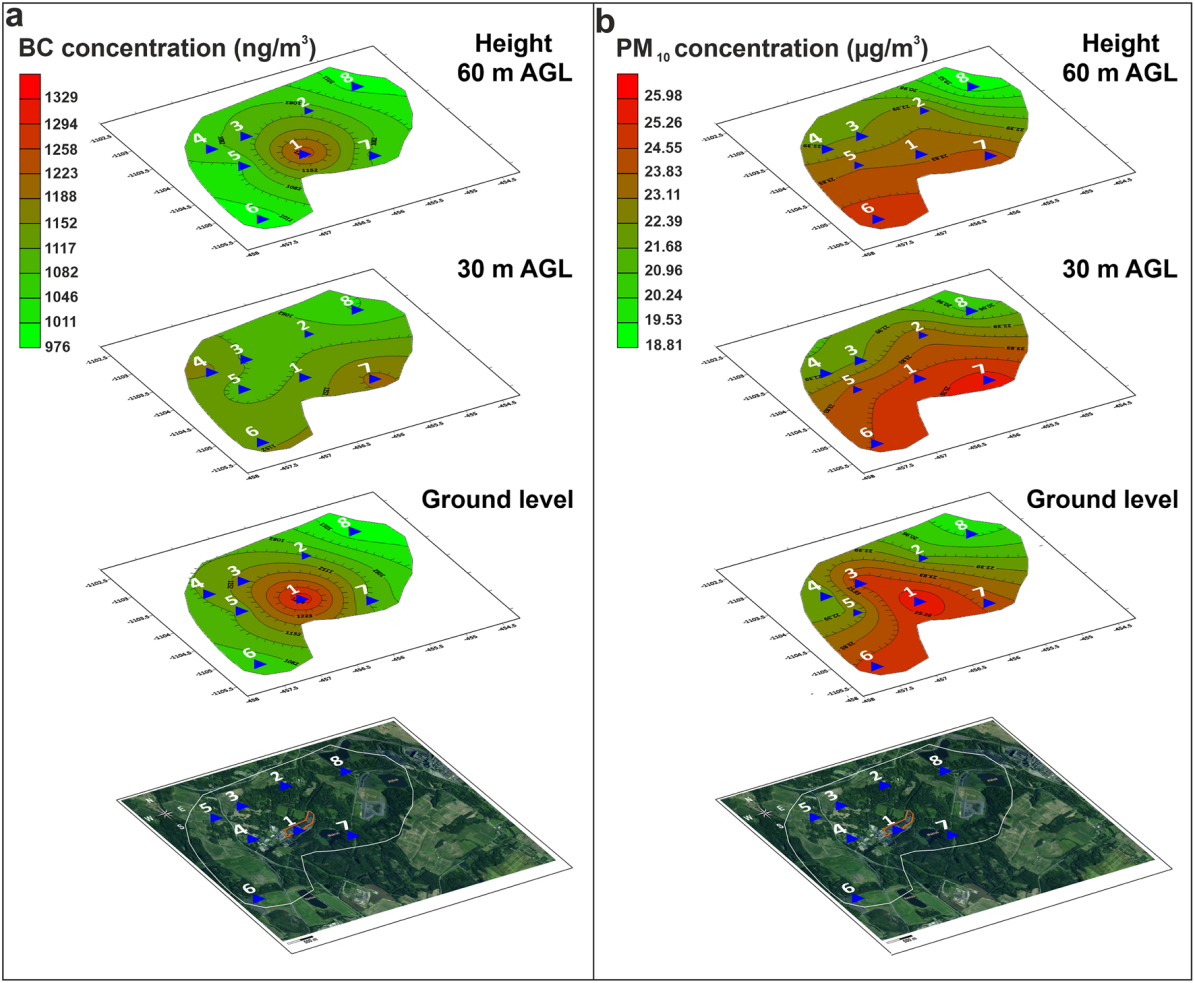
The distribution of BC (**a**) and PM_10_ (**b**) for ground level, 30 m AGL, and 60 m above ground level

The average values for each site (Table 3 and Fig. 6) show no significant dispersion of dust and BC particles from the coal yard. The extent of pollution from the coal yard can be estimated to be between 500 and 1,000 m maximum. Average concentrations of PM_10_ were higher than the centre of the coal yard at 30 m AGL for site No. 7 only (by about 0.86 µg/m^3^) and at 60 m AGL for sites No. 5, 6, and 7. Only average concentrations of PM_10_-TSP were higher at the centre of the coal yard for all measured heights, except for 60 m AGL for site No. 6. The coal yard does not significantly increase the air pollution load in the surrounding area. Higher air pollution loads were recorded at sites Nos. 5 and 6 (Fig. 6). BC particles remain at the source site, in particular at the centre of the coal yard at ground level and at 60 m AGL. The horizontal dispersion of BC is limited to a maximum of 0.5 km. Conversely, PM_10_ particles have a higher area range and spread over a distance of more than 1 km from the source of the pollution. The highest concentrations of PM_10_ are located in the southwest and southeast streams and do not originate from the coal yard.

**Fig. 7:**
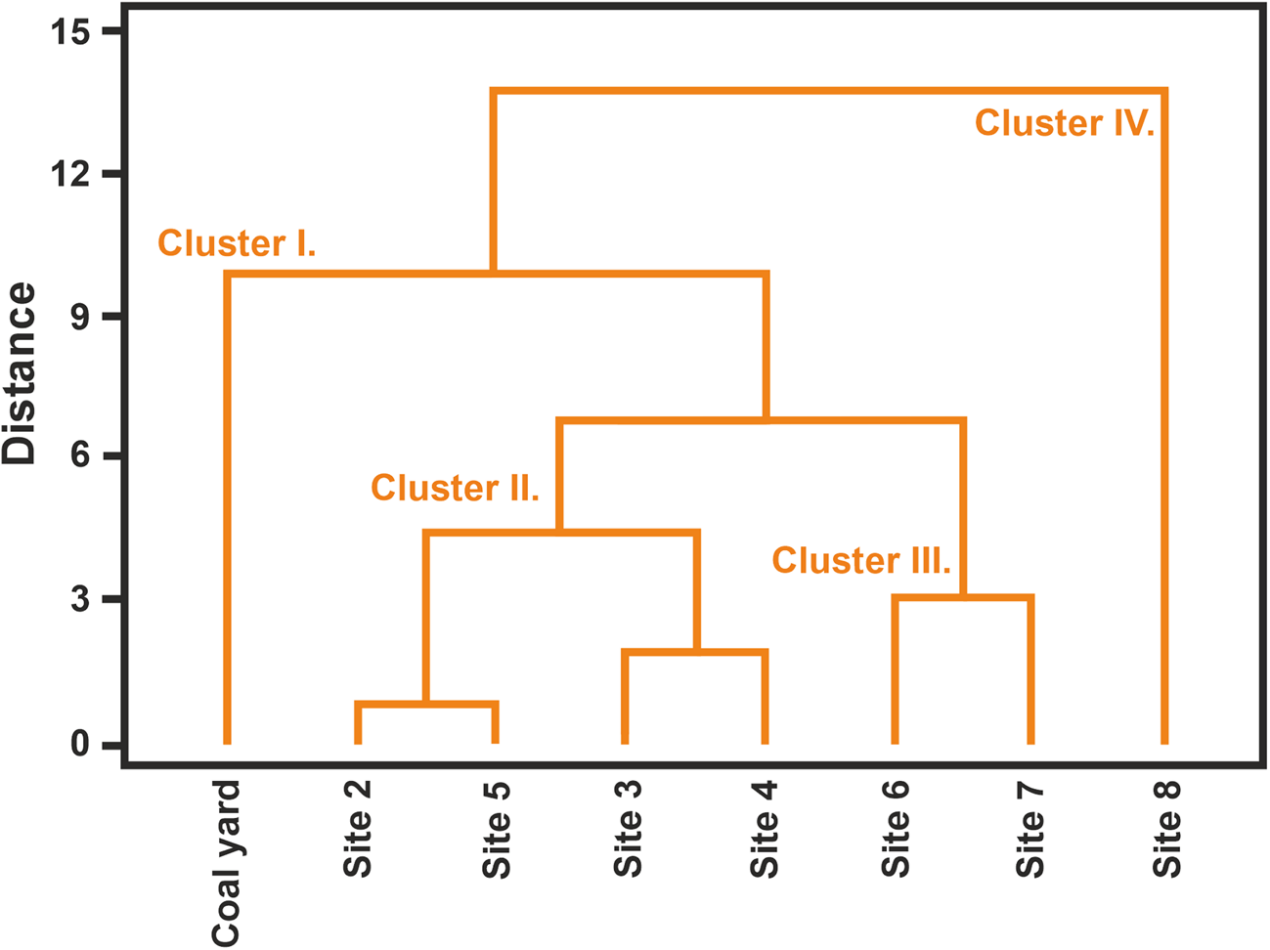
The distribution of localities according to the results of cluster analysis

Based on the distribution of the concentration within the vertical distribution of the air pollutants, the area studied can be divided into sites with higher concentrations and without significant contamination. These results were confirmed by the cluster analysis (Fig. 7), which was created from the values of BC, PM_10_, and PM_10_-TSP concentrations for all the heights studied. The cluster analysis divided the sites into four clusters (Table 5):

- Cluster I, marked as “Coal yard”, showed the highest average values of air pollutants except for PM_10_ at altitudes of 30 m AGL and 60 m AGL.
- Cluster II, named “Area N-NW from the coal yard (sites 2, 3, 4, 5)”, includes sites with the second lowest concentrations of air pollutants. The wind most often flowed from NW, SE, and N directions during the measurements.
- Cluster III, named “Area SE-SW from the coal yard (sites 6, 7)”, includes the second-highest values of PM_10_-TSP and the highest values of PM_10_ at altitudes of 30 and 60 m AGL.
- Cluster IV, “Not affected area”, represents an area without significant load (site 8) with the lowest values of air pollutants at all observed altitudes.

**Table 5:** Average concentrations of BC, PM_10_, and PM_10_-TSP for individual clusters

Cluster	I	II	III	IV
	Coal yard	N-NW from CY	S-SW from CY	Not affected area
BC_GL	1330***	1133	1081**	979*
BC_30 m AGL	1121***	1119.05**	1172	1044*
BC_60 m AGL	1255***	1079	1034**	992*
PM_10__GL	25.99***	22.75**	24.52	19.88*
PM_10__30 m AGL	24.68	22.99**	25.05***	20.59*
PM_10__60 m AGL	23.49	22.87**	24.51***	18.81*
PM_10_-TSP_GL	20.73***	15.83**	17.24	12.37*
PM_10_-TSP_30 m AGL	18.58***	14.19**	16.02	12.77*
PM_10_-TSP_60 m AGL	17.81***	15.05**	16.83	11.33*

Explanations: * – The lowest concentration; ** – The second lowest concentration; *** – The highest concentration

Other sources of pollution near the coal yard and long-distance transport contribute to the resulting pollution around the coal yard.

#### The grain size distribution of dust particles in total suspended particles

**Fig. 8:**
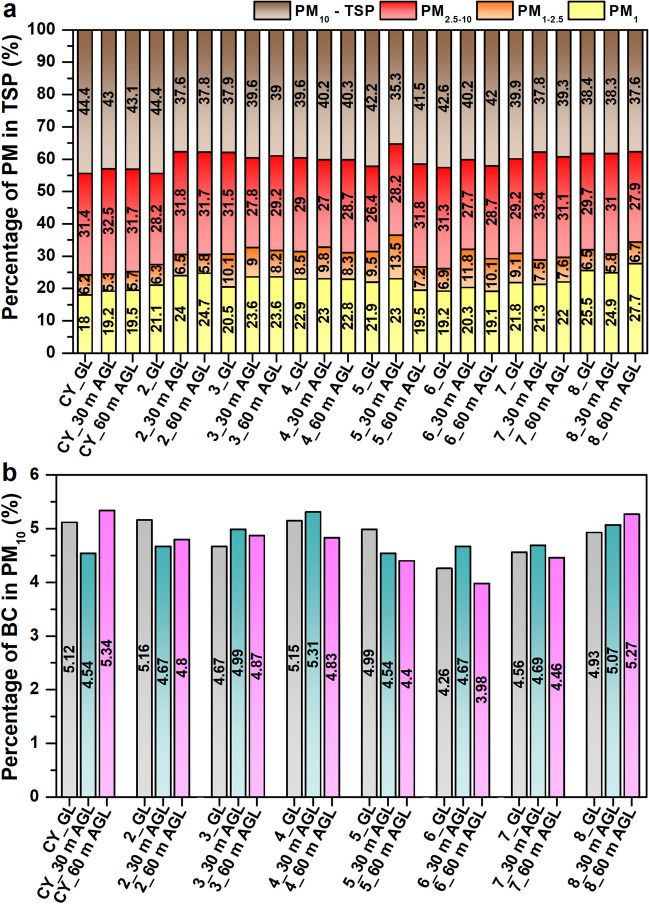
(**a**) Average percentage of particles in each grain class in ∑TSP depending on height for all sites; (**b**) average percentage of BC particles in PM_10_ depending on height for all sites

The average percentage of particles in each grain size class in the ∑TSP for each site and height is shown in Fig. 8. The results of the one-way ANOVA did not confirm significant differences in particle grain size distribution between heights. The amounts of particles PM_<1_ and PM_1-2.5_ are slightly lower above the body of the coal heap than at other sites. In the ∑TSP, coarse-grained particles (PM_10_-TSP) predominate at all monitored sites and heights. The highest average proportion of PM_10_-TSP particles was found for GL, 30 m AGL, and 60 m AGL at the centre of the coal yard (site No. 1-CY).

#### The influence of meteorological parameters on air pollutant concentrations

The amount of BC in PM_10_ above the coal surface and in the vertical profile is also influenced by meteorological conditions. Weather conditions play a key role in influencing the resuspension of coal particles, their stay or transport in the air, and the amount of precipitation. Higher concentrations of PM_10_ particles are observed at low wind speeds, and conversely, PM_10_ decreases at higher wind speeds (Cichowicz et al. 2020). This is due to the greater exchange of the air mass in the assessed area and the displacement of pollutants over greater distances. Also, the current wind direction significantly influences the movement of pollutants from both local and remote sources (Cichowicz et al. 2020). Higher concentrations of BC, TSP, and PM_10_ were observed during rainless days (dry days) than after more heavy rains, which is in line with the results (Zhou et al. 2020).

A statistically significant correlation between the concentration of BC and PM_10_ at all altitudes (1 m, 30 m, and 60 m AGL) was demonstrated in the area studied. PM_10_ show a statistically significant correlation with PM_10_-TSP at all observed altitudes (Table 6). In terms of meteorological parameters, relative humidity has been shown to show a significant correlation with PM_10_ at all observed altitudes. For particles of the grain size class PM_10_-TSP, a significant relative humidity dependence was found only at 30 m AGL and 60 m AGL. Higher concentrations of pollutants were measured at higher relative humidity values. No statistically significant correlation was found between the concentration of BC and relative humidity. This result is probably related to the hygroscopic nature of BC particles. Fresh, young black carbon particles exhibit hydrophobic properties, but during ageing, they undergo a hygroscopic transformation, becoming more capable of absorbing water from the surrounding air (Derouin 2021).

**Table 6:** The values of the correlation coefficient for monitored parameters depending on height

	GL			30 m			60 m		
	BC	PM_10_	PM_10_-TSP	BC	PM_10_	PM_10_-TSP	BC	PM_10_	PM_10_-TSP
PM_10_	0.70*			0.67*			0.72*		
PM_10_-TSP	0.15	0.58*		0.02	0.61*		0.28	0.67*	
Wind speed	-0.44	-0.64*	-0.46	-0.51	-0.57*	-0.47	-0.55	-0.54	-0.31
Wind direction	-0.18	-0.46	-0.69*	-0.01	-0.47	-0.57*	-0.13	-0.33	-0.54*
Relative humidity	0.26	0.63*	0.52	0.25	0.62*	0.72*	0.21	0.53*	0.58*
Temperature	0.36	0.04	-0.61*	0.53	-0.10	-0.63*	0.40	0.01	-0.63*

Explanation: * Correlation is significant at the 0.05 level

Wind speed is another important parameter influencing PM_10_ concentrations. An inverse correlation between wind speed and PM_10_ concentrations was observed at ground level and 30 m AGL. Higher wind speeds were associated with lower PM_10_ concentrations. PM_10_ concentrations at wind speeds ranging from 0.12 to 0.4 m/s were 25.46 ± 7.60 µg/m^3^, while at wind speeds ranging from 0.5 to 0.7 m/s, they were lower (15.83 ± 2.10 µg/m^3^). Air temperature does not significantly affect BC or PM_10_ concentrations, but it does affect PM_10_-TSP concentrations at all observed heights. No correlation between air pollution and air pressure values was demonstrated.

#### ***Daily variation in BC and PM***_***10***_*** concentrations on measurement days***

One-way ANOVA was used to monitor the statistical significance of the differences in daily variability. The significance of the differences in the concentration of different particles (BC, PM_<1_, PM_1-2.5_, PM_2.5–10_, PM_10_-TSP) at heights of 1 m, 30 m, and 60 m AGL for each measurement day was assessed. The analyses did not confirm statistically significant differences in the concentration of particles at heights of 1 m, 30 m, and 60 m AGL on the measurement days.

**Fig. 9:**
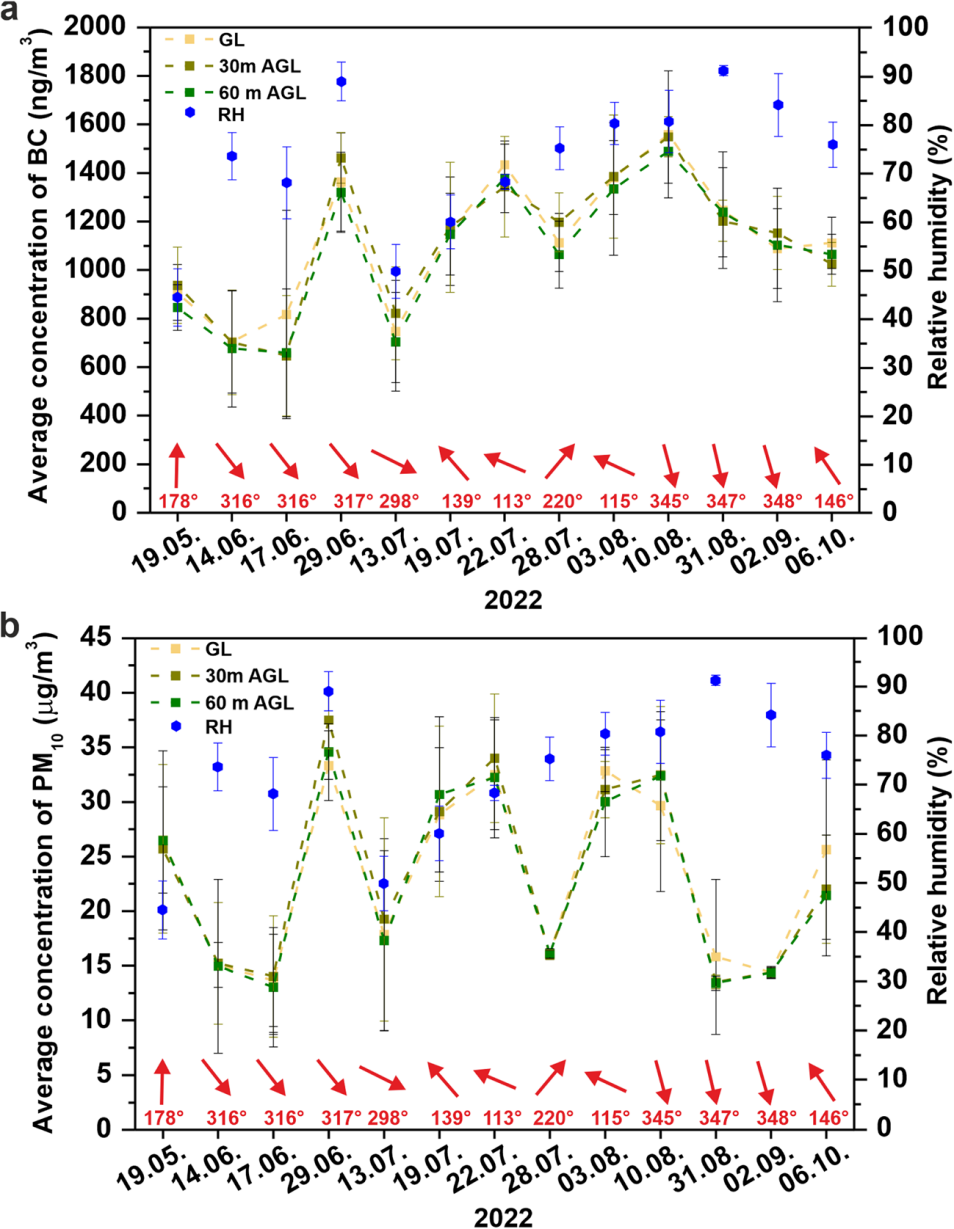
Average daily concentrations at monitored heights for all locations with the indication of relative humidity (RH) and wind direction (**a**) BC; (**b**) PM_10_

Within the daily variation in BC and PM_10_ concentrations, average daily concentrations of BC and PM_10_ (Fig. 9) were generated for the studied area (eight sites). The results of the daily variation in air pollutant concentrations showed that average BC and PM_10_ concentrations were mostly highest at ground level and decreased slightly or remained similar with increasing height.

The highest levels of daily BC concentrations were measured on 10 August 2022 at all monitored altitudes. This day resulted in higher concentrations of PM_10_, especially at altitudes of 30 m AGL and 60 m AGL. Winds from the northern direction probably contributed partially to long-range particle transport, which increased BC and PM_10_ concentrations at these altitudes compared to ground level. The PM_<1_ ratio in ∑TSP was 18.21% for 30 m AGL and 17.14% for 60 m AGL. A weekly rain-free period and a temperature higher than 30 °C contributed to the accumulation of BC in the air.

The lowest average values of BC concentration at all altitudes were found on 14 June 2022 and 17 June 2022. On 17 June 2022, the lowest average values of PM_10_ at ground level and at 60 m AGL were also found. On 17 June 2022, coarse-grained particles from the grain size class 10–100 µm (PM_10_-TSP) predominated in the total amount of TSP, accounting for about 50% of the particles at all observed altitudes. On this day, local sources of emissions had a significant impact on air quality.

On 29 June 2022, the highest PM_10_ concentration values were reached at all monitored altitudes. Approximately one-third of all particles in the ∑TSP were less than 1 µm, and one-third had a size of 10–100 µm. In this case, the particles came from both local sources and long-range transport. On 29 June 2022, the study area recorded one of the lowest average wind speed values (0.15 ± 0.13 m/s) and the second highest relative humidity value (89.00 ± 3.96%). Fine-grained PM_1_ and PM_2.5_ particles are produced by secondary processes during oxidation, ageing or photochemical reactions of primary emissions during the long-range transport of primary pollutants (Kwon et al. 2023). Particularly, PM_1_ particles come from long-range transport (Hien et al. 2021). Based on the results (Hien et al. 2021; Kwon et al. 2023), it can be assumed that the ∑TSP on 31 August 2022 was affected by the long-range transport of particles. The ∑TSP was predominantly formed by fine-grained PM < 1 µm, representing 67.5% at GL level, 90.8% at 30 m AGL, and 92.1% at 60 m AGL.

**Fig. 10:**
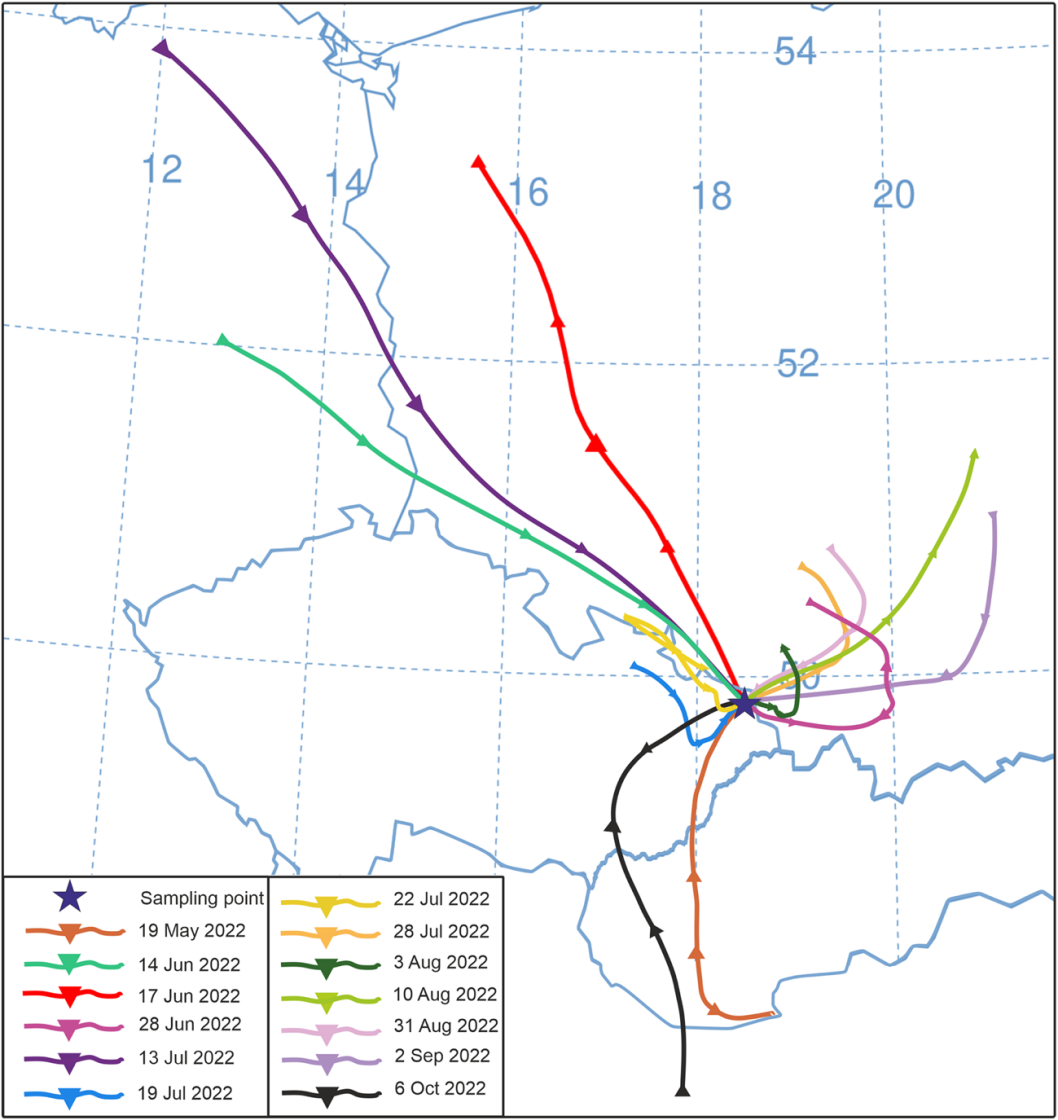
HYSPLIT backward trajectories for individual measurement days in 2022

Daily variability in BC and PM_10_ concentrations does not affect height, but differences occur between measurement days. The situation in the focus area is influenced by both local emission sources and long-range particle transport. Long-range transport accounted for a particularly significant share of the total TSP (∑TSP) content on 28 July 2022, 31 August 2022, and 2 September 2022, with PM_<1_ accounting for the majority of the total TSP content at all monitored heights (from 66.7% to 95.1%). The size distribution of the particles showed that about 1/3 of the period was affected by particles originating from long-distance transport. These results are important for understanding the sources of pollution and can lead to effective measures to protect the environment and the health of the population. The analysis of the backward trajectories performed by HYSPLIT confirmed that the emission load on 29 June 2022, 28 July 2022, 31 August 2022, and 2 September 2022, was influenced by the long-distance transport from the border area of Poland. Almost similar backward trajectories and wind directions were observed on these days (see Fig. 10). On these days, the air masses flowed from relatively short distances from the border area of the Silesian Voivodeship and the peripheral western part of the Lesser Poland Voivodeship in Poland. Silesia, the second most populous and most urbanised region in Poland, is characterised by coal mining and the associated industry, as well as electricity and heat generation. The Upper Silesia region is still the largest coal basin in Europe (Wehner et al. 2017). With the NW and N flow (from the border area of Poland), there is a significant increase in PM_1_ concentrations in the TSP for the study area.

## Conclusion

The use of unmanned aerial vehicles represents an important route for obtaining more accurate information on air pollution and the behaviour of some pollutants that can spread from coal yards. Coal particles are released into the environment during coal handling at the coal yard and as a result of weather conditions. The surface-level distribution model for coal particles shows that the anomaly caused by the operation of the coal yard has a very limited extent. The extent of coal particle pollution from the coal yard ranges from a maximum of 500 to 1,000 m. The spatial distribution was monitored for BC particles at ground level (1 m AGL), 30 m AGL, and 60 m AGL. The average BC concentration decreases with the height above the coal surface. The highest average BC concentration at the centre of the coal yard was measured for the ground level at 1,330 ± 322.8 ng/m^3^. Based on the results found and compared with the difference between the background values, BC particles at the centre of the coal yard were found to be made up of 17% coal particles associated with the activities carried out at the coal yard. The average percentage of coal particles in PM_10_ was almost comparable for measurements at different heights and locations, clearly demonstrating that the coal yard does not affect the air pollution load in the surrounding area. The low elevation dispersion of coal particles confirms the limited pollution range area. Modern approaches using UAV techniques allow for more accurate results and better detection of fugitive emissions within the vertical distribution of coal and dust particles from coal yards. Information on the area and spatial distribution of black carbon in PM_10_ can be used to set concentration limits that will be safe regarding the maximum environmental load.

## Supplementary Information

Below is the link to the electronic supplementary material.

Supplementary file1 (DOCX 13606 KB)

Supplementary file2 (XLSX 47 KB)

## Data Availability

All data generated or analysed during this study are included in this published article.
