# Supplementary figures and images for: Environmental impact assessment of the coal yard and ambient pollution
Source: Environ Sci Pollut Res Int. 2024 Feb 17;32(48):27590–607. doi: 10.1007/s11356-024-32490-z (PMC12695979; doi:10.1007/s11356-024-32490-z)

**Supplementary Material I - HYSPLIT backward trajectories for individual measurement days in 2021.**


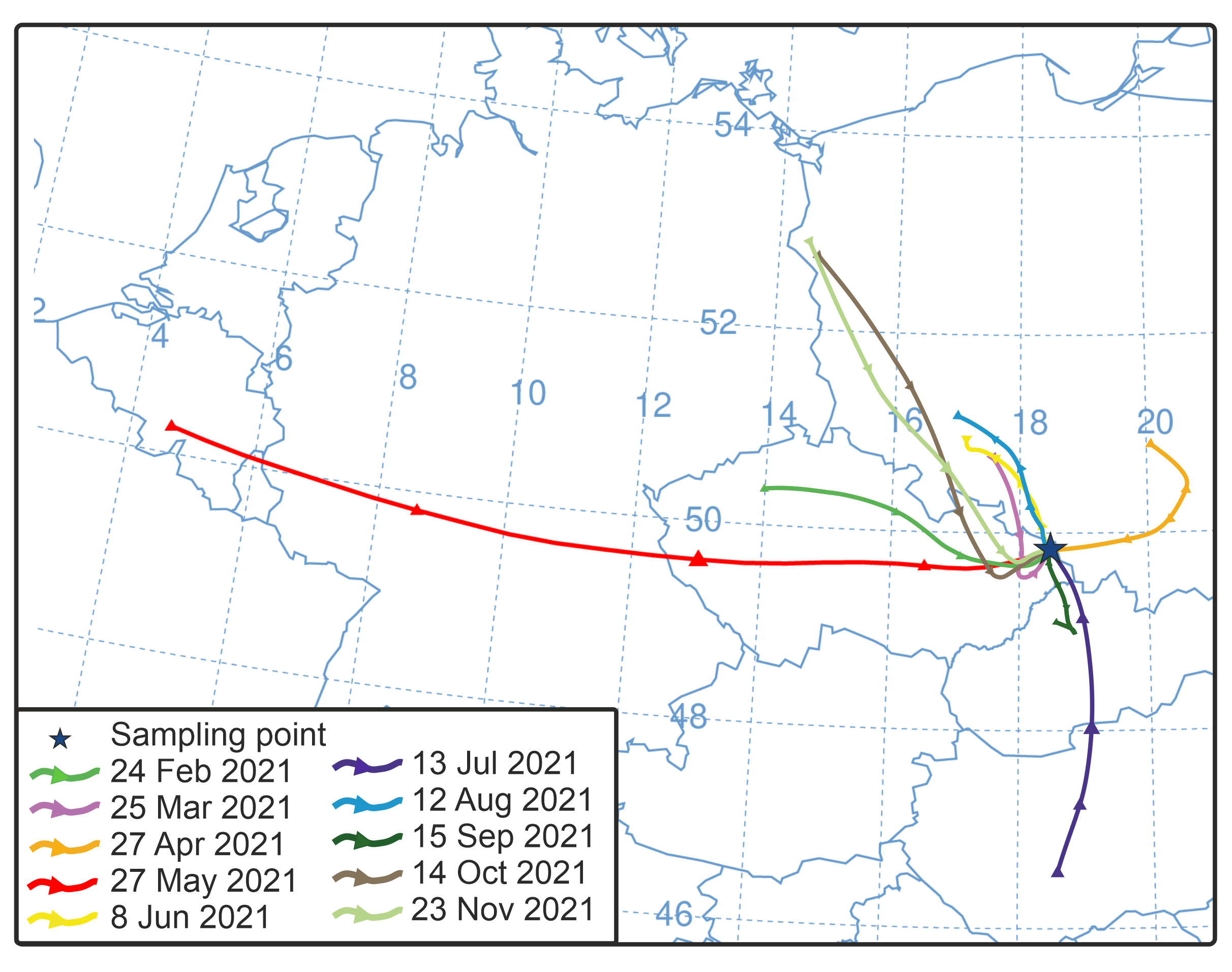

Supplement: Supplementary file 1 — Supplementary file1 (DOCX 13606 KB) [file 11356_2024_32490_MOESM1_ESM.docx]
